# Safe use of PHI6 IN the experimental studies

**DOI:** 10.1016/j.heliyon.2023.e13565

**Published:** 2023-02-08

**Authors:** Enni Sanmark, Joel Kuula, Sirpa Laitinen, Lotta-Maria A.H. Oksanen, Dennis H. Bamford, Nina S. Atanasova

**Affiliations:** a University of Helsinki, Faculty of Medicine, Helsinki, Finland; b Department of Otorhinolaryngology and Phoniatrics – Head and Neck Surgery, Helsinki University Hospital, Helsinki, Finland; c Atmospheric Composition Research, Finnish Meteorological Institute, Helsinki, Finland; d Finnish Institute of Occupational Health, Kuopio, Finland; e Molecular and Integrative Biosciences Research Program, Faculty of Biological and Environmental Sciences, University of Helsinki, Helsinki, Finland

**Keywords:** Surrogate virus, Phi6, Aerosolization, Occupational safety, Bacterial endotoxin

## Abstract

Surrogate viruses theoretically provide an opportunity to study the viral spread in an indoor environment, a highly needed understanding during the pandemic, in a safe manner to humans and the environment. However, the safety of surrogate viruses for humans as an aerosol at high concentrations has not been established. In this study, Phi6 surrogate was aerosolized at high concentration (Particulate matter_2.5_: ∼1018 μg m^−3^) in the studied indoor space. Participants were closely followed for any symptoms. We measured the bacterial endotoxin concentration of the virus solution used for aerosolization as well as the concentration in the room air containing the aerosolized viruses. In addition, we measured how the bacterial endotoxin concentration of the sample was affected by different traditional virus purification procedures. Despite the purification, bacterial endotoxin concentration of the Phi6 was high (350 EU/ml in solution used for aerosols) with both (two) purification protocols. Bacterial endotoxins were also detected in aerosolized form, but below the occupational exposure limit of 90 EU/m^3^. Despite these concerns, no symptoms were observed in exposed humans when they were using personal protective equipment. In the future, purification protocols should be developed to reduce associated bacterial endotoxin levels in enveloped bacterial virus specimens to ensure even safer research use of surrogate viruses.

## Highlights

• Surrogate viruses enable (safe) opportunities to study and model the transmission of different viral pathogens.
• The safety aspects of using bacteriophage Phi6 specimens in aerosol form have not been studied earlier.
• ABacterial endotoxin content was detectable after both purification methods illustrating the future need for removal protocols.
• None of the participants experienced irritation or allergy when using proper personal protective equipment.

## 1 Introduction

Current pandemic, caused by a novel coronavirus SARS-CoV-2, has made us realize how poorly we understand virus transmission routes and their significance especially indoors [1]. Growing evidence highlights the importance of the airborne transmission of SARS-CoV-2 and has increased the debate of the role of airborne transmission for other common epidemic viruses such as norovirus and influenza [[2], [3], [4]]. However, possibilities to investigate the airborne transmission of dangerous pathogens ethically and safely, especially in real-life settings, are weak.

In this study, solid and small enough particles that can travel long distances indoors are defined as aerosols and larger liquid particles settling down near the source are defined as droplets [5,6]. The division is not straightforward and depends significantly on, for example, environmental factors. Even the particles up to 100 μm can remain in the air for longer periods if the settling velocity is exceeded by the velocity of air moving throughout a room [7]. Those particles which meet the definition of an aerosol and carry biological material, such as viruses or bacteria, are called bioaerosols [8].

One possibility to study the airborne transmission outside of the laboratory is to use safer surrogate viruses to model real pathogens. What is a surrogate? All viruses can be divided into a small number of “structure-based” lineages [9]. This data can be used as a guideline in search for apathogenic surrogates that share similar structures than the pathogen of interest. Significant exposure to various bioaerosols has been associated with respiratory symptoms, allergic reactions, and acute infections, but also in some cases with cancer [[10], [11], [12]]. While only a fraction of the total bioaerosol burden is infectious, these non-culturable components such as bacterial endotoxins may still cause allergic reactions, inflammation, sensitization and respiratory symptoms [13], and this has also caused concerns when working with surrogate viruses. The term endotoxin has been used to refer to a biologically active parts of Gram-negative bacteria occurring in the environment [14], mainly lipopolysaccharides (LPS). Endotoxins are released from the outer membrane of bacteria during bacterial lysis or when budding from living bacterial cells. The health risk of bioaerosols can be assessed, for example, by measuring their concentrations and determining the levels of bacterial endotoxins [15,16]. The key to bacterial endotoxin control during viral studies is the purity of the viral specimen.

Bacteriophages are commonly used as surrogates due to the detailed knowledge regarding their structure and host. Phi6, the bacteriophage used in this study, is an enveloped bacterial virus that is not infectious to humans, animals or plants and it has earlier been used as a surrogate to study coronaviruses, mainly in surface and disinfection testing [[17], [18], [19], [20]], but recently also in aerosolized form [21,22]. Phi6 share a common virion architecture (two inner icosahedral shells and an outer lipid envelope with spikes) and a diameter of approximately 80 nm with SARS-CoV-2 [23]. In addition, it can be aerosolized [24]. Although Phi6 is theoretically safe information is based mainly on assumptions about the viral structure and the host bacterium′s inability to infect human cells. On the other hand, the total exposure to for example the bacterial endotoxin load released during the virus production due to lysis of the Gram-negative host bacterium *Pseudomonas syringae* has not been yet properly studied.

Virus purification protocols have traditionally been developed to be able to further analyze samples that contain high concentrations of infectious viruses and minimal amounts of impurities. However, this type of sample purification has not been aimed to produce specimens that would be safe for human exposure, especially during aerosolization, which in virology is considered the highest risk scenario. In our study, we wanted to better understand the potential for safe real-life experiments, the need for which has been demonstrated by the Covid-19 pandemic and the lack of understanding of virus transmission pathways. Surrogate viruses provide a good opportunity for this if they can be used safely with human experiments.

As the need for real-life experiments increases, it is important to know if Phi6 can cause irritation symptoms or respiratory tract or other symptoms for exposed humans if exposure is frequent and/or Phi6 has been aerosolized in large quantities. Our purpose in this study was to determine

1) whether high concentrations of aerosolized Phi6 cause irritation symptoms,
2) whether bacterial endotoxins are detected in the air during Phi6 aerosolization, and
3) whether studied virus purification protocols reduce the bacterial endotoxin content. Based on these our aim was to create the recommendation for the safe use of Phi6.

## 2 Material and methods

This study was conducted as a part of the larger experimental simulation investigating the transmission of aerosolized Phi6 in a restaurant indoor in the restaurant Ultima, Helsinki [25]. During the simulations Phi6 was continuously nebulized from 60 to 90 min in the approximately 60 m^2^ restaurant room. The aerosol temperature right after nebulisation varied between 16 °C and 19 and the ambient room temperature was roughly 19 °C. The relative humidity was approximately 28% [25,26]. Seven out of 11 researchers remained in the room through each simulation. After each simulation symptom questionnaires including large scale of different irritation and allergic symptoms were fulfilled (Supplement 1) [27]. Detailed information of the environment, set-up and study protocol of the experimental simulations is presented in a separate article [25]. Altogether three 90 min and eight 60 min simulations were performed. In addition to the data published by Oksanen et al. which consists of one 90 min simulation and six 60 min simulations, the questionnaire includes two additional 90 min and two 60 min pre-simulations that were performed similarly as described by Oksanen et al. to ensure the functionality of the study protocol [25]. The total duration of the exposure during one-day simulation in this study was always 120–150 min. During simulations, several Phi6 safety measurements were performed. Irritation symptoms were determined each time, but not all other measurements were conducted each time. However, all safety measurements were repeated so that the values could be considered reliable.

### 2.1 Determination of irritation symptoms

After each simulation, all participants completed a questionnaire regarding irritation symptoms including respiratory symptoms, skin symptoms, eye symptoms, and abdominal symptoms. The development of symptoms was monitored for one week after each simulation. Some participants participated in the simulation experiment only once and some in each of the 11 simulation experiments performed in nine separate days.

### 2.2 Media, strains, and viruses

Phi6 bacteriophage and its host bacterium, *Pseudomonas syringae* serovar phaseolicola HB10Y were obtained from Ann Vidaver, University of Nebraska during earlier research of Prof. Bamford [28]. HB10Y was aerobically grown in Luria-Berthani (LB) broth at 22 °C, 23 °C, or 28 °C depending on the experiment (see further).

### 2.3 Phi6 harvesting, purification, and concentration

**Fig. 1:**
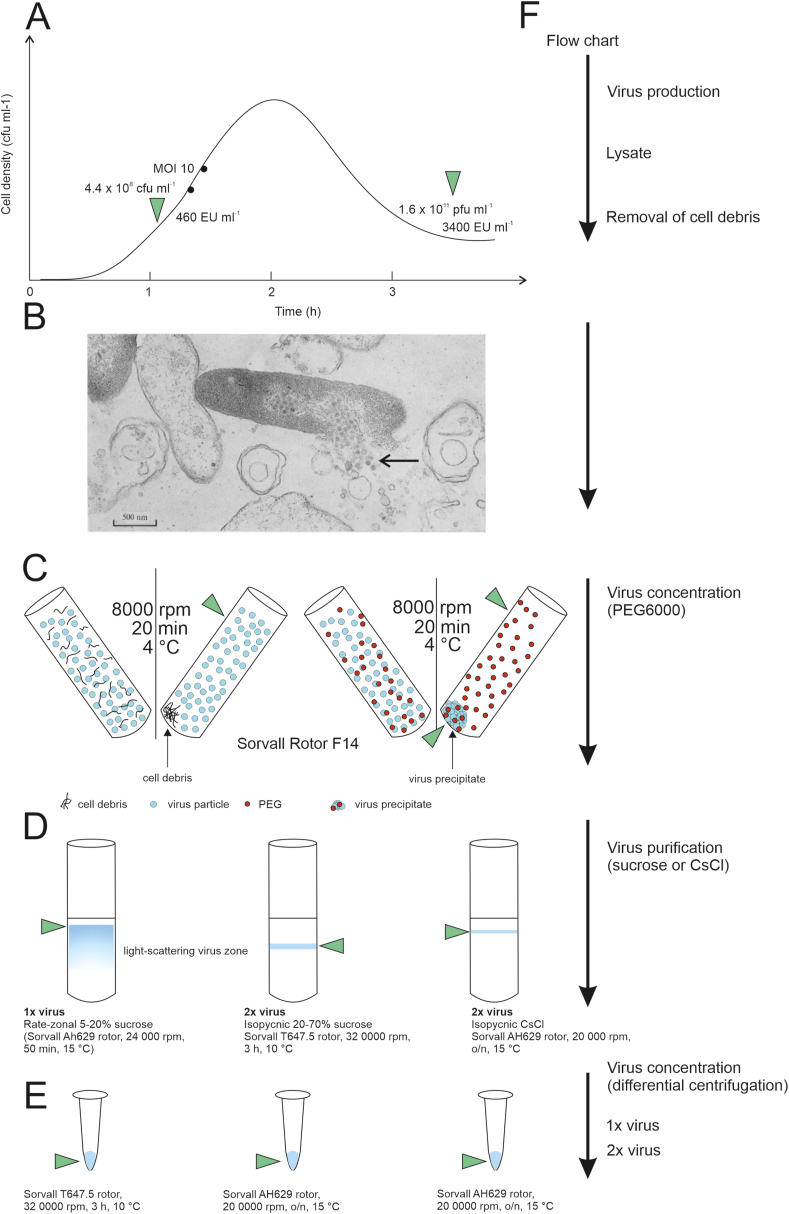
Bacterial endotoxin sampling during virus production and different purification process. Endotoxin sample is indicated with a green triangular arrow. A. Cultivation and infection of *P. syringae* host cells with Phi6 virus. B. Electron micrograph showing the release of Phi6 viruses and endotoxins from infected *P. syringae* cells [42]. Arrow points at released viruses and endotoxins. Used with permission [42]. C. Preparation of virus lysate and virus concentration by PEG6000. D. Virus purification in sucrose or CsCl gradients, rate-zonal or density gradients. E. Concentration of purified viruses by differential centrifugation. F. Experiment flow chart. (For interpretation of the references to colour in this figure legend, the reader is referred to the Web version of this article.)

Four hundred ml of HB10Y cells in LB broth in 2 l side-arm flasks were aerobically grown at 28 °C until optical density at 550 nm wavelength (OD550) of the culture was 0.6. Cells were transferred into 23 °C and grown until OD550 was 0.7. At this stage, the culture was infected with Phi6 with a multiplicity of infection (MOI) 10. The infected culture was grown with 200 rpm shaking at 23 °C for 1 h after which the shaking was reduced to 150 rpm and grown until lysis occurred (D550 value dropped to 0.2). Cell debris was removed by centrifugation (Sorvall Rotor F14, 8000 rpm, 20 min, 4 °C). Harvesting and purification is described more accurate in Fig. 1.

Produced viruses were purified by adding Polyethylene glycol (PEG)6000 and NaCl to reach a final concentration of 10% w/v and 0.5 M, respectively. After applying 1 h magnetic stirring at 4 °C, the precipitate was collected by centrifugation (Sorvall rotor F14, 8000 rpm, 20 min, 4 °C). The precipitate was rinsed using sterile mQ water and suspended to desired volume of 20 mM K-phosphate buffer 1 mM MgCl_2_ overnight at 4 °C on ice. Aggregates were removed by centrifugation (Sorvall rotor F28/50, 10 000 rpm, 10 min, 4 °C) and the supernatant containing the virus particles was purified in 5–20% sucrose gradient in 20 mM K-phosphate buffer by rate-zonal centrifugation (Sorvall AH629 rotor, 24 000 rpm, 50 min, 15 °C). Here, method 1 refers to rate-zonal virus purification and methods 2 and 3 refer to further purification by equilibrium centrifugation (meaning that viruses purified by method 1 are further purified either by method 2 or 3). To obtain purified virus by method 1, the light scattering virus zone was collected and concentrated by differential centrifugation (Sorvall T647.5 rotor, 320,000 rpm, 3 h, 10 °C). Two different methods were used to obtain twice purified virus. In method 2, the light scattering virus zone was further purified in 20–70% sucrose in 20 mM K-phosphate buffer pH 7.2 with 1 mM MgCl_2_ by isopycnic centrifugation (Sorvall AH629 rotor, 20 000 rpm, overnight (o/n), 15 °C) and concentrated as before. In method 3, the light scattering virus zone was purified in CsCl density gradient in 20 mM Tris-HCl buffer, pH 7.2 by isopycnic centrifugation (Sorvall AH629 rotor, 20 000 rpm, o/n, 15 °C). Concentrated viruses were resuspended in desired volume of 20 mM K-phosphate with 1 mM MgCl_2_ pH 7.2 (hereafter referred to as Buffer 1) or 20 mM Tris-HCl buffer with 1 mM MgCl_2_ pH 7.2 (hereafter referred to as Buffer 2) o/n on ice.

### 2.4 Phi6 aerosolization

The Phi6 viruses were aerosolized using an Omron Ultrasonic Nebuliser (model NE-U17, Omron corporation, Japan). The nebuliser uses an ultrasonic vibrator (frequency 1.7 MHz) to form droplets on the surface of the nebulized solution, and a fan pushes the formed aerosol from the nebulisation chamber to the ambient air via a 70 cm long plastic hose (inner diameter 20 mm). The nebulisation rate of the nebulized solution was approximately 0.3 ml/min, and the measured air volume flow rate was approximately 14 l/min. Preliminary laboratory tests with a virus-free buffer solution showed that the nebuliser produced log-normally distributed wet particles whose geometric mean diameter (GMD) was 7 μm. After drying, the size distribution shifted toward smaller particles having a GMD of 0.9 μm. The drying process was observed to last less than a few seconds. With a geometric mean diameter of 7 μm, the calculated nebulisation rate was approximately 3 × 10^8^ particles per second. Phi6 viruses purified by method 1 were diluted in Buffer 1 with a final concentration of infective particles of approximately 1 × 10^10^ pfu/ml. The Phi6 concentration in the nebulized solution was on average 9.1 × 10^9^ pfu/ml, and thus a single 7 μm-sized particle contained approximately 1.6 viruses (calculated as virus concentration multiplied by particle volume). In this study, it was assumed that every measured particle, regardless of its size, contained 1.6 viruses. This is reasoned by the following:

i) Once the particle is formed in the nebulisation process, the number of viruses mixed within a single particle does not change. This is true even though the particle loses most of its volume during the drying process. The virus itself is not volatile.
ii) The initial wet size distribution is log-normally distributed; half of the formed particles contain more viruses and half of the particles contain less viruses than the 7 μm-sized particle. Thus, on average, a single particle contains 1.6 viruses.

### 2.5 Bacterial endotoxin sampling

Samples for endotoxin analysis were collected from different stages of the virus purification process (Fig. 1) in the following way: 10 μl of sample was added into 990 μl of endotoxin-free water. The samples were collected during three different virus purification experiments (methods 1–3) (Table 1). For Test 1, endotoxin samples were taken from purified and concentrated Phi6 virus and Buffer 1. During this test, also the endotoxin level of the Phi6 solution used in the aerosolization was tested. For Test 2, more samples of different purification steps were included in the endotoxin analysis, and for test 3, the purification process was taken even further, and endotoxin samples were collected from viruses purified either by methods 1, 2 or 3. The first endotoxin sample was collected from the HB10Y culture prior to infection (Fig. 1A). Fig. 1B demonstrates cell lysis due to Phi6 egress. Following endotoxin samples were collected from virus lysate, PEG6000 precipitate and supernatant (Fig. 1 C), light-scattering sucrose (method 1), sucrose (method 2) and CsCl (method 3) virus zones (Fig. 1D), and from the concentrated, purified virus samples (Fig. 1E). Virus production, purification and concentration flow chart is shown in Fig. 1F). The three different endotoxin tests referred to in Table 1 are illustrated in Fig. 1 D and E panels.

**Table 1:** Different tests and specimens for bacterial endotoxin analysis.

Test 1: Endotoxins of Phi6 sample purified by method 1	Test 2: Endotoxins of different virus purification steps (method 1 purification process)	Test 3: Endotoxins of different purification processes (methods 1, 2, 3)
Specimen	Specimen	Specimen
Phi6 purified by method 1	Cell culture	Lysate
Buffer 1^a^	Phi6 lysate	sucrose band (method 1, not concentrated)
	PEG supernatant	Purified Phi6, sucrose (method 1)
	PEG pellet	sucrose band (method 2)
	sucrose band	Purified Phi6, sucrose (method 2)
	Phi6 purified by method 1	CsCl band (method 3)
	Buffer 1	Purified Phi6, CsCl (method 3)
	sucrose in Buffer 1	Buffer 2^b^

PEG = polyethylene glycol.

a Buffer 1: 20 mM K-phosphate pH 7.2 mM MgCl.

b Buffer 2: 20 mM Tris-HCl pH 7.2 1 mM MgCl_2_.

### 2.6 Bacterial endotoxin analysis

Inhalable endotoxins were collected into a glass fiber filter (1.0 μm, Ø 25 mm, SKC Inc.) by an IOM Sampler at a flow rate of 2 l/min. The biologically active endotoxins in the air and in the Phi6 solution were measured using a validated kinetic chromogenic Limulus Amebocyte Lysate assay (Kinetic QCL, Lonza). The results were expressed as endotoxin units (EU) per cubic meter of air (EU/m^3^) or EU/ml of the Phi6 solution.

### 2.7 Aerosol concentration measurements

Within the restaurant, real-time aerosol concentrations were measured using two model 3321 Aerodynamic Particle Sizers (APS, TSI Inc., USA). APS is a time-of-flight-based aerosol spectrometer, which measures the aerodynamic size of particles from 0.5 to 20 μm with a 52-bin resolution. The time resolution of the measurements was 10 s. The APS instruments were equipped with total suspended particle (TSP) inlets, and the sampling was conducted approximately on the same height as the human airways (0.5 m above table level). One of the APS’s was placed near the nebuliser (APS_near_) and the other one on the other side of the room (APS_far_), approximately 7 m away from the nebuliser. The set-up is presented more detailed in our previous article [25].

#### 2.7.1 Estimated inhalation dose

**Fig. 2:**
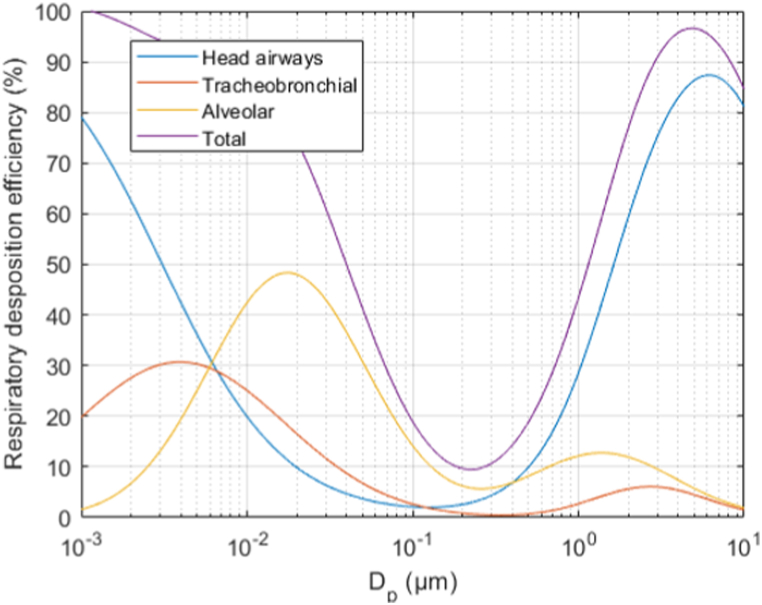
Respiratory deposition efficiency of 0.001–10 μm sized particles (ICRP, 1994) [29].

The Phi6 dose inhaled by the experiment subjects was calculated from the particle size-resolved APS data using the assumption of 1.6 viruses per particle and by using a lung deposition model presented by the International Commission on Radiological Protection [29]. According to ICRP, the deposition efficiency of different sized particles to the human respiratory system can be estimated using the function curves shown in Fig. 2 [29]. These curves describe the fraction (%) of particles, which deposit to the head airways, tracheobronchial, and alveolar region (4) of a human respiratory system as a function of particle size. The total deposition efficiency is the sum of the regional depositions. Mathematical equations for these functions have also been presented by Hinds [30] (1999). The impact of using a respirator (FFP2 or FFP3) was also considered. The dose inhaled was calculated as follows:

$$Inhalation\;dose=\sum_{i=1}^{52}\left((1-{Resp}_{leak}\right){*V}_{inhaled}*(1-{Resp}_{flt})*\;n_{i}*1.6*D_{eff,i})+({Resp}_{leak}*\;V_{inhaled}*\;n_{i}*1.6*D_{eff,i})$$

where ${Resp}_{leak}$ is the respirator internal leak, $V_{inhaled}$ is the inhaled (or sampled) air volume, ${Resp}_{flt}$ is the respirator filtration efficiency, $n_{i}$ is the particle number concentration of the ith size bin and $D_{eff,i}$ is the deposition efficiency of particles of ith size bin. The APS measured particle number concentrations in 52 size bins spaced logarithmically from 0.5 to 20 μm.

### 2.8 Used personal protective equipment

Seven persons participated in each simulation experiment. Because of the lack of knowledge about sufficient personal protective equipment (PPE) all participants dressed in fabric overall, nitrile gloves, foot covers, hair cover, FFP3 respirators (N99) or FFP2 respirators (N95) and protective eyewear. FFP2 respirators were used during three (150 min each) simulation experiments and FFP3 respirators during last eight experiments (60 min each). The change in the use of masks was because of the improved availability. PPE was used during the whole simulation experiment and also 30 min after the experiment when working in the same space where the experiment was performed.

### 2.9 Ethical aspects

All procedures that involved human participants, including environmental sampling, were conducted in accordance with the ethical standards of the institutional or national research committee and the 1964 Declaration of Helsinki and its later amendments or comparable ethical standards. The Ethics Committee of Helsinki University Hospital approved the study protocol (HUS/1701/2020). All respondents provided informed consent prior to their participation.

## 3 Results

### 3.1 Irritation symptoms

A total of 11 different individuals (researchers) participated in the simulation experiments. 7/11 (64%) were female and the mean age of participants was 39,1 ± 9,2 (range 25–51) years. Two persons participated in each of eleven simulation (inside the room during the simulation) experiments and two persons participated only once, other seven participated into 2–10 simulations. None of the participants reported any irritation symptoms (burn/itching/redness of eyes 0%, itchy skin 0%, rash 0%, mouth or throat symptoms 0%, shortness of breath 0%, cough 0%, runny nose 0%, nasal congestion 0%, stomach symptoms 0%, anaphylaxis 0%.).

### 3.2 Bacterial endotoxin concentrations

Bacterial endotoxin concentrations of the different Phi6 virus specimens are shown in Table 2. Endotoxin level of the HB10Y cell culture is 460 EU/ml indicating that some autolysis or budding from living cells is taking place, albeit at low level. The lysis of the cells by Phi6 elevates the endotoxins to 3400 EU/ml. PEG precipitation concentrates the viruses from 1.6 E11 pfu/ml to 8.4 E12 pfu/ml and simultaneously endotoxins from 3400 EU/ml to 33 000 EU/ml indicating that a lot of endotoxins are concentrated along with the viruses. PEG supernatant contains only 3.2 E8 pfu/ml and 2500 EU/ml of endotoxins indicating that there is free endotoxin rather than cell-bound endotoxin that associated with the viruses. However, the endotoxin concentration is low (160 EU/ml) in the sucrose virus band while virus titer remains high (9.9 E11 pfu/ml). When the viruses in the band are concentrated by differential centrifugation to 2E14 pfu/ml, the endotoxin level rises to 87 000 EU/ml. The endotoxin concentrations of the purified viruses are highlighted in Table 2 with bolded table borders. The endotoxin concentration of the Phi6 solution (1 × virus diluted 1:100 into Buffer 1) used for aerosolization was 350 EU/ml.

**Table 2:** Bacterial endotoxin concentrations of the different Phi6 virus specimens.

Test 1: Endotoxins of Phi6 sample purified by method 1			Test 2: Endotoxins of different virus purification steps (method 1 purification process)			Test 3: Endotoxins of different purification processes (methods 1, 2, 3)		
Sample	Endotoxins (EU/ml)	Titer (pfu/ml)	Sample	Endotoxins (EU/ml)	Titer (pfu/ml)	Sample	Endotoxins (EU/ml)	Titer (pfu/ml)
Purified virus (method 1)	81 000	4.3 e14	Cell culture	460	4.4e8 cfu/ml	Lysate	3400	3.9e11
Buffer 1	4.4	ND^a^	Phi6 lysate	3400	1.6e11	sucrose band (method 1)	250	1.1e12
			PEG supernatant	2500	3.2e8	Purified virus (method 1)	92 000	1.4e14
			PEG pellet	33 000	8.4e12	sucrose band (method 2)	290	5.7e11
			sucrose band	160	9.9e11	Purified virus (method 2)	53 000	9.5e13
			Purified virus (method 1)	87 000	2.e14	CsCl band	<50	1.0e12
			Buffer 1	<0.05	ND	Purified virus (method 3)	85 000	7.2e13
			sucrose in Buffer 1	<0.05	ND	Buffer 2	<0.5	ND

a ND, not determined. SD = standard deviation.

The endotoxin concentration measured directly from air during aerosolization was 72 EU/m^3^ at a distance of 0.3 m and 12 EU/m^3^ at a distance of 1.3 m from the nebuliser.

### 3.3 Aerosol concentrations

During the experiments, the measured aerosol number concentrations ranged between ∼3 and 2000 cm^−3^ with the APS_near_ showing the highest concentrations. These concentrations can be considered high as the respective maximum PM_2.5_ concentrations translate to ∼1018 μg m^−3^, when assuming a particle density of 2.3 g cm^−3^ (corresponds approximately to the density of K_3_PO_4_ and MgCl_2_ used in Buffer 1 solution). Unlike the preliminary laboratory experiments, the GMDs of the number size distributions were slightly smaller, approximately 0.7 μm for the APS_near_ and 0.6 μm for the APS_far_ (Table 3).

**Table 3:** Calculated exposure dose for FFP2 and FFP3 respirators based on the amount of Phi6 aerosols measured with APS.

			FFP2^a^	FFP3^b^	No mask
Average	APS_near_	Head airways	2.12E+07	4.66E+06	1.57E+08
		Tracheobronchial	1.79E+06	3.95E+05	1.32E+07
		Alveolar	1.22E+07	2.69E+06	6.01E+07
		Total	3.52E+07	7.75E+06	2.60E+08
	APS_far_	Head airways	1.12E+07	2.47E+06	8.28E+07
		Tracheobronchial	9.90E+05	2.18E+05	7.32E+06
		Alveolar	5.81E+06	1.28E+06	4.29E+07
		Total	1.80E+07	3.97E+06	1.33E+08
Std	APS_near_	Head airways	2.90E+06	6.36E+05	2.10E+07
		Tracheobronchial	2.72E+05	5.99E+04	2.01E+06
		Alveolar	1.25E+06	2.79E+05	4.34E+07
		Total	4.35E+06	9.66E+05	3.25E+07
	APS_far_	Head airways	1.22E+06	2.71E+05	9.04E+06
		Tracheobronchial	1.13E+05	2.49E+04	8.31E+05
		Alveolar	5.47E+05	1.19E+05	4.07E+06
		Total	1.87E+06	4.14E+05	1.40E+07

a Filtration efficiency 94%, internal leak 8%.

b Filtration efficiency 99%, internal leak 2%.

## 4 Discussion

In this study we investigated whether aerosolized Phi6 causes irritation symptoms to subjects exposed to high concentrations of viral aerosols. In addition, we investigated can we decrease endotoxin levels when we purify Phi6 twice with two different optimized purification methods. We found that despite the high concentration of viral aerosols and high bacterial endotoxin levels, we did not observe irritation symptoms in any participants after any of the simulations, neither after repeated exposures. Bacterial endotoxin levels were not significantly reduced with optimized virus purification indicating that endotoxin and virus molecules are not separated by classical virus purification protocols.

There are no earlier studies on the irritation symptoms or safety of aerosolized surrogate viruses. In contrast, other, more commonly found bioaerosols (for example, pollen or organic dust to which compost workers are exposed to) have been found to cause both allergic symptoms and sensitization at high exposures [31,32]. Surrogate viruses are similar in size to ordinary viruses with the size between 20 and 300 nm [33] When we know that particles <500 nm are small enough to penetrate deep in the respiratory tract during breathing, aerosolized Phi6 is easily transported to the lower respiratory tract during inhalation [33]. Although the particles are small enough to reach the lower respiratory tract, the most common irritation symptoms after the exposure to bioaerosols are the irritation of the upper airway or the eyes [34]. We did not determine whether Phi6 causes symptoms without respirators or protective eyewear because of the high loads of bioaerosols, but when using minimum FFP2 respirator and eye protection, irritation was not noted. The result is limited by the small number of participants (n = 11). However, the large number of repetitions (11 simulations) and repeated exposures (11 exposures with two subjects and only two single exposures) increase the reproducibility of the results. In the future, when aerosolizing Phi6, it would still be good to continue the collecting information about the safety aspects.

Bacterial endotoxins are the most common contaminants of purified biological specimens [35]. European Pharmacopoeia requirements state that endotoxin levels in pharmaceuticals for intravenous use should be below 5 EU/1 kg body weight and in pharmaceutical water below 0.25 EU/ml. LPS forms micelles in aqueous solution and interacts with different types of proteins forming aggregates, including virus capsid proteins, for this reason, it is difficult to remove from biological specimens [36]. The different virus purification procedures did not significantly result in the decrease of endotoxin concentration in the final samples (methods 1–3, Table 3). This implies that although viruses are successfully purified from cell debris and environmental impurities by these optimized protocols, the endotoxins derived from the host cell membrane still associate with the final purified virus sample. Accordingly, in the production of the final concentrated and purified virus samples (Fig. 1D), the endotoxins were concentrated along the viruses (Table 3). Both the viruses and the endotoxins were concentrated approximately 1000-fold (Table 3). It is possible that for example the high g force during differential centrifugation leads to an increase of endotoxins from remaining cell debris. Thus, additional methods are required to achieve a purified virus specimen devoid of endotoxin.

Kondratova et al. successfully removed LPS from adeno associated virus (AAV) specimens using mild detergent treatment [35]. Due to its micellar form, detergent breaks down LPS. However, virus infectivity is sensitive to detergents, especially in the case of enveloped viruses, such as Phi6 (AAV is non-enveloped virus). Thus, detergent treatment is not necessarily an optimal method for LPS removal from enveloped virus specimens that are required to remain highly infectious, such as the purified Phi6 that was used in our earlier study [25]. Future studies are needed to address the infectivity of enveloped viruses after detergent treatment. Different chromatography methods have also been used in the removal of endotoxins from biological specimens [37] and because chromatography, especially asymmetrical flow field-flow fractionation [38], can also be used to purify viruses, after careful optimization regarding the affinity of specific virus proteins to endotoxin molecules, it could represent a useful way to prepare virus samples that are safer to use in aerosol studies.

However, it needs to be notified that the final virus samples from purification process are highly concentrated samples with extremely high concentration of infectious viruses. When the sample is used for example in aerosolization or other experiments, it is always diluted, often with several magnitudes. Thus, the bacterial endotoxin level of the aerosolized solution (350 EU/ml) is already significantly lower, and the endotoxin concentrations in the air were below the exposure limit of 90 EU/m^3^. Data from endotoxin exposure assessments indicate that adverse health effects are not expected until after exposure at above 90 EU/m^3^ (NEC and DECOS, 2011). In the future, if we desire to provide a Phi6-purified virus specimen with a lower endotoxin content, purification must be optimized in an endotoxin-removing manner.

The aerosol concentration during experiments with Phi6 viruses was remarkably high. Exposure to aerosols were at the same level than in microorganisms and aerosol-emitting facilities such as in livestock farming and waste processing [39]. At those workplaces exposure to bioaerosols is associated with a wide range of health effects including e.g. respiratory symptoms and lung function impairment. Therefore, we expected an adverse health risk potential of Phi6 aerosols for persons who participated in the simulation experiments. However, the use of respirators resulted in a significant reduction in the number of bioaerosols entering the airways. It is good to note that respirators lose part of their theoretical maximum protective effect if the fit is not perfect, and leakage is usually observed between the respirator and the face (face-seal leakage) [40]. Indeed, our results are encouraging that, despite this, no respiratory or other allergy or irritation symptoms were observed, even with repeated or prolonged exposures. Still, due to the high bioaerosol concentration, we recommend choosing a well-fitting respirator when aerosolizing the Phi6 virus.

A limitation in the experimental setup of this study was the lack of continuous monitoring environmental variables (temperature, relative humidity, or pressure). Environmental parameters were not measured continuously during the experiments and therefore their effect on the aerosol particles was not studied. In general, of the environmental factors, relative humidity could have an impact on the results as it in part drives the drying – and thus the size – of the nebuliser-generated wet aerosol particles. Intermittent measurements showed that the relative humidity within the room was approximately 28%. It is unlikely that this variation had an impact on the measurements because the drying process is much faster (a few seconds maximum) [41], than the experiment duration (60–90min). This means that the particle size distribution reached dry steady state relatively fast and probably did not skew the size distribution measurements in a significant way. High bioaerosol concentrations require generally the use of a respirator. However, respirators do not filter all bioaerosol (approximately at least 94%), and that is why it is the important result that, even during long, repeated exposure, a high bioaerosol concentration did not cause irritation or allergy symptoms to the test subjects. This enables a more comprehensive study of airborne viruses even at high bioaerosol concentrations.

## 5 Conclusion

Surrogate viruses such as Phi 6 have high value as a model that enables the study of viruses harmful to humans (such as SARS-CoV-2) in real-life experiments. However, their safety aspects regarding high exposures especially when aerosolized has not been studied earlier. This study provides further insight into the safety of the Phi 6 virus when used in aerosolized form in the real-life environment and presents bacterial endotoxin levels after different purification processes. Despite the high bioviral aerosol and endotoxin levels, none of the participants in the simulations experienced irritation or allergy symptoms when using personal protective equipment including respiratory protection. Although no allergy symptoms were observed, from the point of view of bioaerosols, it is recommended to use overall, gloves, hair cover, well-fitting FFP2 or FFP3 respirators and protective eyewear.

## Author contribution statement

Enni Sanmark: Conceived and designed the experiments; Performed the experiments; Analyzed and interpreted the data; Contributed reagents, materials, analysis tools or data; Wrote the paper. Joel Kuula: Performed the experiments; Analyzed and interpreted the data; Contributed reagents, materials, analysis tools or data; Wrote the paper.

Lotta Oksanen: Conceived and designed the experiments; Performed the experiments; Contributed reagents, materials, analysis tools or data; Wrote the paper.

Sirpa Laitinen: Conceived and designed the experiments; Performed the experiments; Contributed reagents, materials, analysis tools or data; Wrote the paper.

Nina Atanasova: Conceived and designed the experiments; Performed the experiments; Analyzed and interpreted the data; Contributed reagents, materials, analysis tools or data; Wrote the paper.

Dennis Bamford: Conceived and designed the experiments; Analyzed and interpreted the data; Contributed reagents, materials, analysis tools or data; Wrote the paper.

## Funding statement

This work was supported by the Helsinki University Hospital Research Fund, Finska Läkaresällskapet (ES), Jalmari and Rauha Ahokas foundation (ES), 10.13039/501100014438Business Finland Corona Co-Creation fund Project No. 40 988/31/2020 and Co-Innovation Project No. 3884/31/2021 (ALL), and 10.13039/501100002341Academy of Finland COVID-19 special funding 335681 (NSA).

## Data availability statement

Data will be made available on request.

## Declaration of interest’s statement

The authors declare that they have no known competing financial interests or personal relationships that could have appeared to influence the work reported in this paper.
