# Supplementary material for: Safe use of PHI6 IN the experimental studies
Source: Heliyon. 2023 Feb 8;9(2):e13565. doi: 10.1016/j.heliyon.2023.e13565 (PMC9984441; doi:10.1016/j.heliyon.2023.e13565)
Supplement: Multimedia component 1 [file mmc1.docx]

QUESTIONNAIRE ABOUT POSSIBLE SYMPTOMS AFTER EVERY SIMULATION

**BASICS:**

Date:

Name:

Age:

Gender:

**ESPOSURE AND PROTECTIVE MEASURES:**

Exposure time:

Used protective measures ( Yes/No): mask (type), gloves, hat, overalls

**SYMPTOMS (yes/no):**

Eyes: burn / itching / redness

Skin: itching/ rash

Mouth/Pharynx: mouth or throat symptoms

Pulmonary symptoms: shortness of breath /cough/ runny nose / nasal congestion

Others: stomach symptoms / anaphylaxis

Time when symptoms started (during simulation, how long after simulation?)

Describe the symptoms in more detail in your own words:
